# Microrna expression signatures predict patient progression and disease outcome in pediatric embryonal central nervous system neoplasms

**DOI:** 10.1186/s13045-014-0096-y

**Published:** 2014-12-31

**Authors:** Maria Braoudaki, George I Lambrou, Krinio Giannikou, Vasilis Milionis, Kalliopi Stefanaki, Diane K Birks, Neophytos Prodromou, Aggeliki Kolialexi, Antonis Kattamis, Chara A Spiliopoulou, Fotini Tzortzatou-Stathopoulou, Emmanouel Kanavakis

**Affiliations:** University Research Institute for the Study and Treatment of Childhood Genetic and Malignant Diseases, University of Athens, “Aghia Sophia” Children’s Hospital, Athens, Greece; First Department of Pediatrics, University of Athens, Hematology and Oncology Unit, Choremeio Research Laboratory “Aghia Sophia” Children’s Hospital, Athens, Greece; Department of Medical Genetics, University of Athens, Athens, Greece; Department of Pathology, Children’s Hospital “Aghia Sophia”, Athens, Greece; Department of Neurosurgery, Anschutz Medical Campus, University of Colorado, Denver, CO USA; Department of Neurosurgery, Children’s Hospital “Aghia Sophia”, Athens, Greece; Department of Forensic Medicine and Toxicology, School of Medicine, University of Athens, Athens, Greece; First Department of Pediatrics, University of Athens Medical School, Choremeio Research Laboratory, Thivon & Levadias, 11527, Goudi, Athens, Greece

**Keywords:** Medulloblastomas, Atypical teratoid/rhabdoid tumors, Embryonal tumors, MicroRNA microarrays, Prognosis, Biomarkers

## Abstract

**Background:**

Although, substantial experimental evidence related to diagnosis and treatment of pediatric central nervous system (CNS) neoplasms have been demonstrated, the understanding of the etiology and pathogenesis of the disease remains scarce. Recent microRNA (miRNA)-based research reveals the involvement of miRNAs in various aspects of CNS development and proposes that they might compose key molecules underlying oncogenesis. The current study evaluated miRNA differential expression detected between pediatric embryonal brain tumors and normal controls to characterize candidate biomarkers related to diagnosis, prognosis and therapy.

**Methods:**

Overall, 19 embryonal brain tumors; 15 Medulloblastomas (MBs) and 4 Atypical Teratoid/Rabdoid Tumors (AT/RTs) were studied. As controls, 13 samples were used; The First-Choice Human Brain Reference RNA and 12 samples from deceased children who underwent autopsy and were not present with any brain malignancy. RNA extraction was carried out using the Trizol method, whilst miRNA extraction was performed with the mirVANA miRNA isolation kit. The experimental approach included miRNA microarrays covering 1211 miRNAs. Quantitative Real-Time Polymerase Chain Reaction was performed to validate the expression profiles of miR-34a and miR-601 in all 32 samples initially screened with miRNA microarrays and in an additional independent cohort of 30 patients (21MBs and 9 AT/RTs). Moreover, meta-analyses was performed in total 27 embryonal tumor samples; 19 MBs, 8 ATRTs and 121 control samples. Twelve germinomas were also used as an independent validation cohort. All deregulated miRNAs were correlated to patients’ clinical characteristics and pathological measures.

**Results:**

In several cases, there was a positive correlation between individual miRNA expression levels and laboratory or clinical characteristics. Based on that, miR-601 could serve as a putative tumor suppressor gene, whilst miR-34a as an oncogene. In general, miR-34a demonstrated oncogenic roles in all pediatric embryonal CNS neoplasms studied.

**Conclusions:**

Deeper understanding of the aberrant miRNA expression in pediatric embryonal brain tumors might aid in the development of tumor-specific miRNA signatures, which could potentially afford promising biomarkers related to diagnosis, prognosis and patient targeted therapy.

**Electronic supplementary material:**

The online version of this article (doi:10.1186/s13045-014-0096-y) contains supplementary material, which is available to authorized users.

## Background

Central nervous system (CNS) pediatric tumors of embryonal origin are a heterogeneous group of malignant neoplasms that comprise by far the largest group of malignant brain tumors in childhood. All embryonal tumors are classified pathologically as grade IV malignancies and they are largely comprised of medulloblastomas (MBs) and atypical teratoid/rhabdoid tumors (AT/RTs), among others [1]. Medulloblastoma is considered the most common malignant pediatric brain tumor, accounting for 20% of cases [2]. Risk stratification based on clinical parameters is insufficient for accurate prognostication [3]. Current treatment options include surgery, chemotherapy and radiotherapy [4]. However, age limitations for the delivery of radiotherapy have been set due to the vulnerability of the developing brain to radiotherapy-induced neurocognitive deficits [5]. Notwithstanding, aggressive multimodal therapy has improved the prognosis for children with MB, nearly one third of patients will eventually succumb to progressive tumors. Atypical teratoid/rhabdoid tumour is also a highly malignant CNS tumour that represents 1-2% of pediatric brain tumours and accounts for at least 10% of CNS tumours in infants, due to the predominance in children younger of 3 years old. The survival of children younger than 3 years of age remains poor, particularly for patients with supratentorial tumors and those with metastatic disease [2]. It can be supratentorial, especially in cerebral hem [6] ispheres, or infratentorial, especially in the cerebellar hemispheres, cerellopontine angle and brain stem mainly in children younger than 2 years of age. Prognosis of AT/RT is dismal, while there are no protocols aimed specifically for this type [2].

The mainstay treatment for both local and distant disease control in older children, normally >3 years remains craniospinal irradiation [7]. Nevertheless, outcomes remain highly associated with increased mortality due to current therapy-resistant disease. It is therefore likely that improved treatments will only be possible when the tumor-specific molecular events are better understood.

Growing evidence suggests that a class of small cellular RNAs, termed microRNAs (miRNAs) play important roles in controlling the development of the CNS, by regulating cell proliferation, differentiation and apoptosis [8,9]. Recent miRNA-based research reveals that a) miRNA profiles of tumor cells are dissimilar from normal cells and b) miRNA expression profiles in tumors from similar developmental origins have similar alterations [10]. Understanding the interplay between normal brain development and CNS tumors pathogenesis is essential for the development and implementation of more efficient and less toxic targeted therapies [4]. Given these, and the fact that miRNAs are less susceptible to chemical modification and RNase degradation, provide the rationale that miRNAs might afford substantial value for neuro-oncology. Thus far, approaches to miRNA-targeted therapies have been mentioned in lung cancer, lymphoma and pancreatic cancer [11-14]. Regarding brain tumors, to the best of our knowledge, there are no reports available of miRNA use for therapeutic purposes, except for *in vitro* systems [15,16]. In particular, the use of miR-34a in several cancer models is encouraging, since it has been found down-regulated in diverse types of tumors [17-22] and its expression might suppress the ontogenesis of those specific types of malignancies.

Undeniably, thus far, expression profile analysis has revealed several miRNA signatures related to malignant pediatric brain tumors [9,23-27]. However, as the number of novel miRNAs is still increasing, large-scale screening is necessary to profile the global miRNA expression. To our knowledge, only a few studies applying miRNA microarrays have been conducted [24,28,29] in pediatric brain tumors. MiRNA microarrays afford the most commonly used tool for the large-scale screening of miRNA expression. Subsequently, bearing in mind that a) miRNAs represent one of the largest classes of gene regulators and b) the clinical significance of miRNA expression profiles as well as their biological role in pediatric CNS embryonal tumors remains to be elucidated, characterization of miRNA patterns in pediatric malignant MBs and AT/RTs might have substantial value for diagnostic and prognostic purposes as well as for advanced therapeutic interventions.

In the current setting we performed for the first time miRNA microarrays and meta-analyses based on miRNA microarrays datasets from GEO to identify and validate miRNA candidates relevant to pediatric CNS embryonal prognosis. The obtained miRNA patterns were correlated to several factors known to affect survival including tumor type, age at diagnosis and tumor recurrence. We found that certain miRNA profiles were relevant to each of the aforementioned variables and their possible combinations delivering potential reliable diagnostic, prognostic or therapy-related targets, while this is the first report that underlines the adverse role of miR-34a and its association with embryonal brain tumor patients’ inferior prognosis.

## Results

### MicroRNA expression profiling between tumor groups

**Figure 1:**
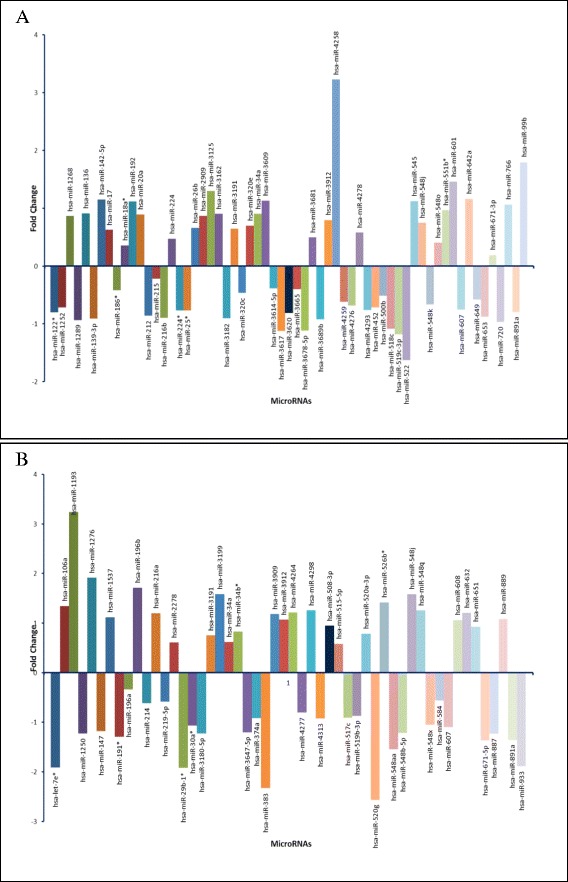
**Histogram graphical representations of the differentially expressed miRNAs between embryonal tumors and the control group.** Comparisons between MBs and the control group **(A)**. Comparisons between the AT/RTs and the control subjects **(B)**. Y axis values represent miRNA fold change. Overall, 113 miRNAs were found differentially expressed.

In the current study, we identified a total of 113 DE miRNAs (*p < 0.05* and FDR < 0.05) in the embryonal tumor group (MBs, *n*_*miRNA*_ = 61 and AT/RTs, *n*_*miRNA*_ = 52) when compared to the non-malignant group of patients (Figure 1). Additionally, following meta-analysis 1242 DE miRNAs were identified (*p < 0.05* and FDR < 0.007).

**Figure 2:**
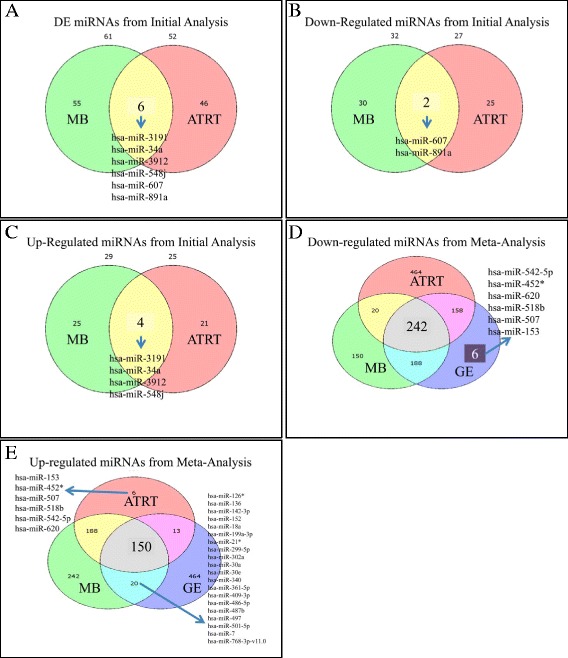
**Overlapping relationship of the differentially expressed miRNAs.** Venn diagrams illustrate the overlapping relationship of the number of up-regulated miRNAs among MBs and AT/RTs in initial and meta-analysis data. In particular, 6 DE miRNAs were common in MB and ATRT samples from the initial experiments **(A)**. Two were common in Down-regulated miRNAs between both MBs and ATRTs **(B)**, as well as four were common in up-regulated miRNAs between MBs and ATRTs **(C)**. Further, 242 common down-regulated miRNAs were identified between MBs, ATRT and GE samples in all experimental datasets (meta-analysis) **(D)**. Additionally, six miRNAs appeared to be unique in Germinoma samples **(D)**. Finally, 150 miRNAs were identified to be common in up-regulated MB, ATRT and GE samples from the meta-analysis results **(E)**. Additionally, six miRNAs appeared to be unique in ATRT samples, within the same analysis, as well as 20 miRNAs were common between MB and GE samples **(E)** (Legend: MB: Medulloblastoma, ATRT: Atypical Teratoid/Rhabdoid Tumor, GE: Germinoma).

Overall, the majority of the 113 DE miRNAs initially observed were down-regulated with a total of 54 miRNAs (47.8%) exhibiting increased expression and 59 miRNAs (52.2%) showing decreased expression. Among them, 107 miRNAs were characterized as tissue-specific, whilst 6 miRNAs; miR-34a, miR-548j, miR-607, miR-891a, miR-3191 and miR-3912 were found to be consistently differentially expressed in both MBs and AT/RTs (Figure 2A). Of note, the majority of them were also found differentially expressed in both MBs and AT/RTs, following our meta-analyses. Specifically, miR-34a was found overexpressed in all embryonal tumor samples tested when compared to the control group, following both our initial and meta-analyses (including GEs) (Figure 2A, 2C, 2E). Further on, miR-548j, miR-3191 and miR-3912 were found overexpressed by our initial analysis only (Figure 2C), whereas miR-607 and miR-891a were found down-regulated in all embryonal tumor samples tested as compared to the control group, following our initial analysis only (Figure 2B). The overlapping relationship of the DE miRNAs among both tumor types following initial and meta-analyses is depicted in Figure 2. In addition, all commonly down- or up-regulated miRNAs are presented in Additional file 1: Table S2.

**Figure 3:**
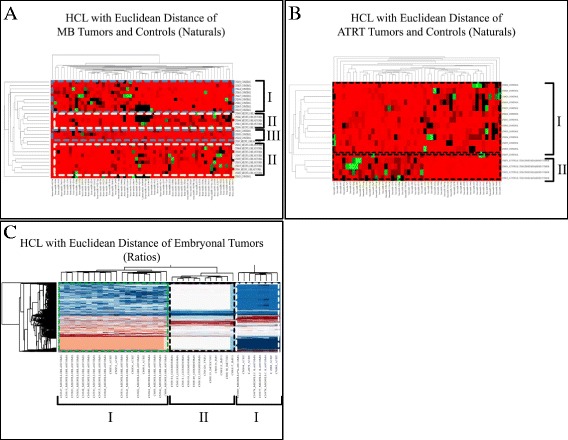
**Hierarchical clustering (HCL).** MiRNA profiling did not discriminate between MBs and the control group following initial analysis **(A)**. MiRNA profiling accurately discriminated between AT/RTs and the control group following initial analysis **(B)**. Similarly, meta-analysis discriminated between Embryonal tumors and GEs, yet by creating two groups of embryonal tumors **(C)**.

Unsupervised two-way hierarchical clustering (HCL) with Euclidian distance confirmed that normal samples and AT/RTs cluster independently, however it did not discriminate accurately between MBs and the normal control group (Figure 3). Following our initial analysis, miRNA profiling did not discriminate between MBs and the control group (Figure 3A). However, it appeared that differences occurred regarding samples from cerebellum and medulla oblongata (Additional file 2: Table S1). MiRNA profiling accurately discriminated between AT/RTs and the control group (Figure 3B). Similarly, meta-analysis discriminated between GEs and the rest embryonal tumors (Figure 3C).

### MicroRNA expression and patient age

Following our initial analysis, we evaluated differences regarding patient age at diagnosis. Patients were divided into three groups based on the therapeutic protocol they received; Group A: < 3 years (*n* = 8), Group B: 3–8 years (*n* = 4) and Group C: 9–18 years (*n* = 7). More specifically, Group A did not receive any radiation therapy, whereas Group B and Group C received mild and more intense radiation therapy, respectively. MiRNA expression associations were made among all aforementioned individual patient groups. Following our initial analysis, overall, 11 differentially expressed miRNAs were identified, including miR-1268, miR-2052, miR-26b, miR-3665, miR-3681, miR-3912, miR-519c-3p, miR-601, miR-608, miR-720 and miR-891a. Among them, 6 miRNAs; miR-1268, miR-3681, miR-3912, miR-601, miR-608 and miR-720 were overexpressed in Group A, 3 miRNAs; miR-3665, miR-519c-3p and miR-891a were overexpressed in Group B and 2 miRNAs; miR-2052 and miR-26b were overexpressed in Group C. In addition, 3 miRNAs; miR-519c-3p, miR-3665 and miR-891a were down-regulated in Group A, 6 miRNAs; miR-1268, miR-2052, miR-26b, miR-3912, miR-601 and miR-608 in Group B, whereas 2 miRNAs; miR-720 and miR-3681 were down-regulated in Group C (Additional file 3: Figure S2).

For the meta-analyses, individuals were age categorized as following: **Fetal**; samples in gestation period (n = 76), **Infants**; for individuals aged <1 year old (n = 19), **Children**; for individuals >1 and <11 years old (n = 42), **Adolescents**; for individuals >11 years and <18 years old (n = 6) and **Adults**; for individuals >18 years old (n = 5). Of note, using this type of analysis, similar results were obtained. More specifically, all the aforementioned differentially expressed miRNAs obtained, following the initial analysis were once more found differentially expressed after correlations with the new age categories. The only miRNAs that were not validated were miR-3681 and miR-608. All results are summarized in Additional file 4: Table S3.

### MicroRNA expression and gender

Overall, 8 miRNAs were found differentially expressed when comparisons were performed between miRNA expression levels and gender. Specifically, 3 miRNAs; miR-26b, miR-3162 and miR-1268 were consistently up-regulated in males, whilst 5 miRNAs including miR-720, miR-186*, miR-3617, miR-320c and miR-3614-5p were found overexpressed in females (Additional file 5: Figure S3). Following meta-analysis, only miR-122* was found differentially expressed between males and females. This result was consistent for both the ratio values (tumors/controls) and the natural values (no ratio, no log2 transformation). The results are summarized in Additional file 6: Table S3.

### MicroRNA expression and disease progression

MicroRNA expression was compared between three groups: control group, patient group in complete remission (CR) and relapsed (RE) patient group. In total, 18 miRNAs were found differentially expressed (Additional file 7: Figure S4). Based on the current findings, 8 miRNAs; miR-3681, miR-601, miR-320e, miR-34a, miR-642a, miR-136, miR-26b and miR-192 were found overexpressed in the patient group. Among them, 3 miRNAs were found up-regulated in the RE patient group and 5 miRNAs were found overexpressed in the CR patient group. In addition, 10 miRNAs; miR-720, miR-891a, miR-522, miR-518c, miR-3665, miR-3620, miR-382, miR-452, miR-122 and miR-147 were found down-regulated in the patient group, indicating tumor suppressor properties. Following meta-analysis, miR-122, miR-3162 and miR-642a were found to be significantly different between RE and CR samples in the log_2_, ratio transformed expression values. Especially, miR-122 expression appeared to follow a descending trend from MB to ATRT to GE. The miR-122 tumor suppressor property was confirmed by meta-analysis (Table 1). Additionally, accounting for the natural values, miR-122 was found to be significantly different between control and RE samples as well as RE and CR samples. Regarding miR-3162 expression, it followed an ascending trend from MB to ATRT to GE. MiR-3162 and miR-642a were also found to be significantly different in associations between RE and CR samples. Of note, the oncogenic property of miR-642a as detected following the initial analysis, was not confirmed by meta-analysis (Table 1). The overexpression of miR-601 initially detected as well as the down-regulation of miR-147 initially observed were not validated. The results are summarized in Additional file 6: Table S3.

**Table 1:** **Summary of miRNA expression signatures as prognostic parameters**

**MicroRNAs**	**Disease progression high → low**	**Clinical outcome**	**MiRNA property (Initial analysis)**	**MiRNA property (Meta-analysis)**	**Prognosis**
		**High → low**			
**miR-3681**	CR/R/C	A/D/C	Oncogene	Oncogene	Inferior
**miR-601**	CR/R/C	A/D/C	Oncogene	Tumor-suppressor gene	Favorable
**miR-320e**	R/CR/C	D/A/C	Oncogene	Tumor-suppressor gene	Favorable
**miR-34a**	R/CR/C	N/A	Oncogene	Oncogene	Inferior
**miR-642a**	CR/R/C	A/D/C	Oncogene	Tumor-suppressor gene	Favorable
**miR-720**	C/CR/R	C/A/D	Tumor-suppressor gene	Tumor-suppressor gene	Favorable
**miR-891a**	C/R/CR	C/D/A	Tumor-suppressor gene	Tumor-suppressor gene	Favorable
**miR-522**	C/R/CR	N/A	Tumor-suppressor gene	Oncogene	Inferior
**miR-518c**	C/CR/R	N/A	Tumor-suppressor gene	Oncogene	Inferior
**miR-3665**	C/CR/R	N/A	Tumor-suppressor gene	Tumor-suppressor gene	Favorable
**miR-3620**	C/CR/R	N/A	Tumor-suppressor gene	Tumor-suppressor gene	Favorable
**miR-382**	C/CR/R	N/A	Tumor-suppressor gene	Tumor-suppressor gene	Favorable
**miR-452**	C/CR/R	N/A	Tumor-suppressor gene	Tumor-suppressor gene	Favorable
**miR-136**	R/CR/C	A/D/C	Oncogene	Oncogene	Inferior
**miR-26b**	CR/R/C	A/D/C	Oncogene	Oncogene	Inferior
**miR-122**	C/CR/R	N/A	Tumor-suppressor gene	Tumor-suppressor gene	Favorable
**miR-147**	C/CR/R	N/A	Tumor-suppressor gene	Oncogene	Inferior
**miR-192**	R/CR/C	N/A	Oncogene	Oncogene	Inferior

High → Low expression, CR; clinical remission, R; relapse, C; control, A; alive, D; deceased, P; positive, N/A; not applicable.

**Table 2:** **Clinical and demographic data of the patient cohort used in initial analysis**

	***N***	**All**	**Males**	**Females**	**Medulloblastoma**	**ATRT**	**Germinoma**	**Controls**	**Mean age (years)**	**STDEV age**	**Mean age (weeks)**	**STDEV age**
**Initial analysis**	**ALL**	32							6.15	4.74	331.14	227.61
	**MALES**	22							8.41	4.98	439.46	238.84
	**FEMALES**	10							4.12	3.42	233.65	164.39
	**UNKNOWN GENDER**	0							NaN	NaN	NaN	NaN
	**MEDULLOBLASTOMA**	15	8	7					7.13	4.62	378.41	221.92
	**ATRT**	4	1	3					2.46	3.06	153.90	146.95
	**GERMINOMA**	0	0	0					NaN	NaN	NaN	NaN
	**CONTROLS**	13	13	0					NaN	NaN	NaN	NaN
	**FETAL**	0	0	0	0	0	0	0	NaN	NaN	NaN	NaN
	**INFANT**	3	1	2	1	2	0	0	0.34	0.30	52.53	14.18
	**CHILD**	13	5	8	11	2	0	0	5.80	3.44	314.59	165.35
	**ADOLESCENT**	3	3	0	3	0	0	0	13.45	1.85	681.48	88.71
	**ADULT**	0	0	0	0	0	0	0	NaN	NaN	NaN	NaN
	**%**	**ALL**	**MALES**	**FEMALES**	**MEDULLOBLASTOMA**	**ATRT**	**GERMINOMA**	**CONTROLS**				
	**ALL**	100.00%										
	**MALES**	68.75%										
	**FEMALES**	31.25%										
	**UNKNOWN GENDER**	0.00%										
	**MEDULLOBLASTOMA**	46.88%	4.97%	4.35%								
	**ATRT**	12.50%	0.62%	1.86%								
	**GERMINOMA**	0.00%	0.00%	0.00%								
	**CONTROLS**	40.63%	8.07%	0.00%								
	**FETAL**	0.00%	0.00%	0.00%	0.00%	0.00%	0.00%	0.00%				
	**INFANT**	9.38%	0.62%	1.24%	0.62%	1.24%	0.00%	0.00%				
	**CHILD**	40.63%	3.11%	4.97%	6.83%	1.24%	0.00%	0.00%				
	**ADOLESCENT**	9.38%	1.86%	0.00%	1.86%	0.00%	0.00%	0.00%				
	**ADULT**	0.00%	0.00%	0.00%	0.00%	0.00%	0.00%	0.00%

**Table 3:** **Clinical and demographic data of the patient cohort used in the Meta-analysis**

	***N***	**All**	**Males**	**Females**	**Unknown gender**	**Medulloblastoma**	**ATRT**	**Germinoma**	**Controls**	**Mean age (years)**	**STDEV AGE**	**Mean Age (weeks)**	**STDEV age**
**Meta-analysis**	**ALL**	161								4.15	11.91	226.32	574.91
	**MALES**	82								3.85	13.76	210.26	663.33
	**FEMALES**	46								1.09	2.43	76.16	122.39
	**UNKNOWN GENDER**	32								9.20	14.00	476.79	672.56
	**MEDULLOBLASTOMA**	19	8	7	4					6.89	4.13	366.95	198.43
	**ATRT**	8	1	3	4					2.23	2.18	142.95	104.49
	**GERMINOMA**	12	0	0	12					11.15	3.08	571.00	147.76
	**CONTROLS**	121	73	36	12					3.04	13.43	169.45	647.52
	**FETAL**	76	40	32	4	0	0	0	76	0.00	0.00	18.39	3.41
	**INFANT**	19	15	4	0	1	2	0	16	0.56	0.54	62.67	25.71
	**CHILD**	40	9	10	21	15	6	9	10	5.82	3.52	315.53	168.84
	**ADOLESCENT**	6	3	0	3	3	0	3	0	14.07	1.57	711.54	75.34
	**ADULT**	5	2	0	3	0	0	0	5	56.48	29.58	2746.94	1419.60
	**%**	**ALL**	**MALES**	**FEMALES**	**UNKNOWN GENDER**	**MEDULLOBLASTOMA**	**ATRT**	**GERMINOMA**	**CONTROLS**				
	**ALL**	100.00%											
	**MALES**	50.93%											
	**FEMALES**	28.57%											
	**UNKNOWN GENDER**	19.88%											
	**MEDULLOBLASTOMA**	11.80%	4.97%	4.35%	2.48%								
	**ATRT**	4.97%	0.62%	1.86%	2.48%								
	**GERMINOMA**	7.45%	0.00%	0.00%	7.45%								
	**CONTROLS**	75.16%	45.34%	22.36%	7.45%								
	**FETAL**	47.20%	24.84%	19.88%	2.48%	0.00%	0.00%	0.00%	47.20%				
	**INFANT**	11.80%	9.32%	2.48%	0.00%	0.62%	1.24%	0.00%	9.94%				
	**CHILD**	24.84%	5.59%	6.21%	13.04%	9.32%	3.73%	5.59%	6.21%				
	**ADOLESCENT**	3.73%	1.86%	0.00%	1.86%	1.86%	0.00%	1.86%	0.00%				
	**ADULT**	3.11%	1.24%	0.00%	1.86%	0.00%	0.00%	0.00%	3.11%				

### MicroRNA expression and clinical outcome

MicroRNA expression was compared between three groups: control group, patient group-survivors (alive) and patient group-deceased. In total, 8 miRNAs were associated with patients’ clinical outcome; miR-3681, miR-601, miR-320e, miR-642a, miR-720, miR-891a, miR-136 and miR-26. Specifically, 2 miRNAs; miR-720a and miR-891a were down-regulated and 6 miRNAs; miR320e, miR-3681, miR-601, miR-642a, miR-136 and miR-26b were overexpressed in the patient cohort when compared to the control group. Based on the current findings, among the overexpressed miRNAs, only miR320e was related to inferior prognosis as it was significantly up-regulated in the deceased group when compared to the group of patients that remain alive. On the contrary, the majority of the rest miRNAs were associated with favorable prognosis, since they were found to be consistently up-regulated in patients that remain alive when compared to the deceased group of patients (Additional file 8: Figure S5). Following meta-analyses, all 8 deregulated miRNAs that were identified initially were also found deregulated in previous associations with disease progression. However, once more, the overexpression of miR-601 initially detected was not validated, suggesting tumor suppressor activities. In addition, the oncogenic properties initially detected for miR-642a and miR-320e were not confirmed with meta-analyses suggesting tumor suppressor activities. However, the oncogenic role of miR-3681, miR-26b and miR-136 were validated with meta-analyses (Table 1). The results are summarized in Additional file 6: Table S3.

### qRT-PCR validation

**Figure 4:**
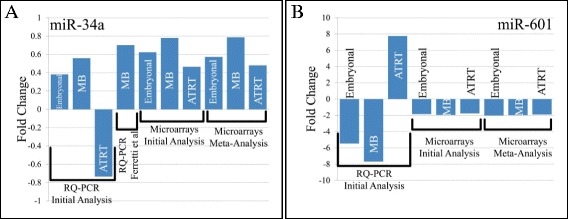
**Comparative diagram of Real-Time expression data with microarray expression.** Comparison of Real-Time fold change of embryonal, MB and AT/RT samples by initial analysis, by Ferretti et al. microarray fold change by initial and meta-analyses for miR-34a **(A)** and miR-601 **(B)**. Where: MB; Medulloblastoma, AT/RT; Atypical Terhatoid/Rhabdoid Tumor).

We verified by qRT-PCR the expression levels of miR-34a. According to our results, miR-34a was found up-regulated in all samples tested including the embryonal tumors group and the MB group alone, following both initial and meta-analyses. In addition, using the qRT-PCR raw data from Ferretti et al. once again the up-regulation of miR-34a in pediatric MBs was verified. Of note, the overexpression of miR-34a was not validated in the AT/RT group alone following initial analysis (Figure 4A). Regarding miR-601 initial analysis and meta-analyses did not coincide as far as AT/RTs were concerned. The qRT-PCR results revealed similar expression patterns with meta-analyses regarding the embryonal tumors group and the MB group alone (Figure 4B).

### ROC analysis

**Figure 5:**
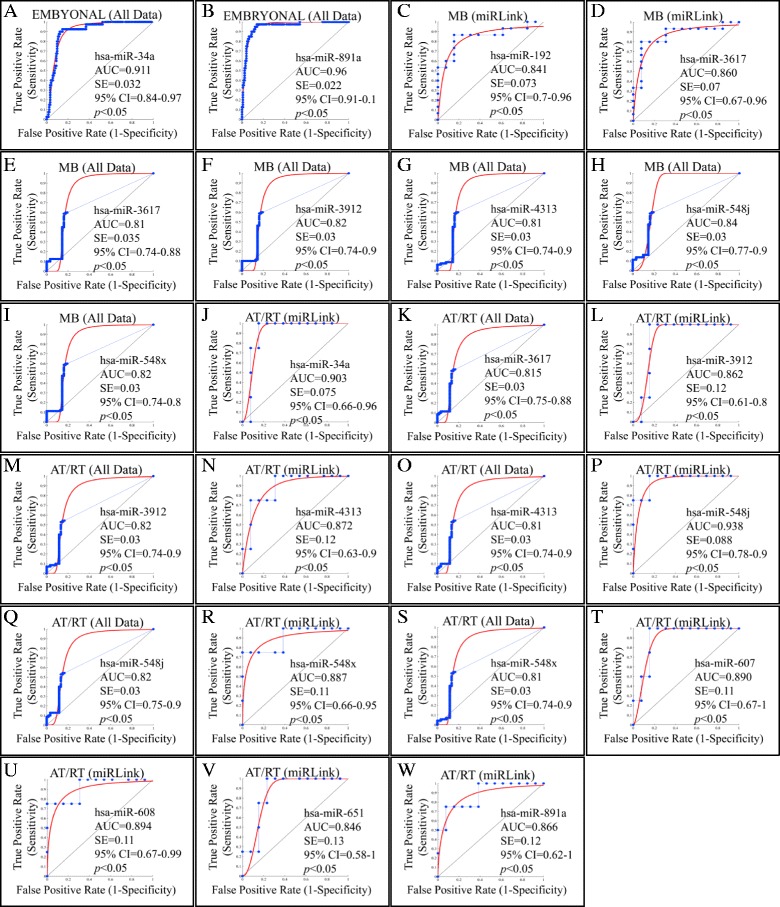
**ROC analysis of miRNA data.** ROC curves of the differentially miRNAs in each tumor type using the microarray expression data. Embryonal tumors could be separated by miR-34a **(A)** and miR-891a **(B)** following meta-analysis. MBs could be separated by miR-192 **(C)**, miR-3617 **(D)** following initial analysis, whereas miR-3617 **(E)**, miR-3912 **(F)**, miR-4313 **(G)**, miR-548j **(H)** and miR-548x **(I)** following meta-analysis. AT/RT samples could be separated by miR-34a following initial analysis **(J)**, miR-3617 following meta-analysis **(K)**, miR-3912 following initial analysis **(L)**, miR-3912 following meta-analysis **(M)**, miR-4313 following initial-analysis **(N)**, miR-4313 following meta-analysis **(O)**, miR-548j following initial analysis **(P)**, miR-548j following meta-analysis **(Q)**, miR-548x following initial analysis **(R)**, miR-548x following meta-analysis **(S)**, miR-607 following initial analysis **(T)**, miR- following initial analysis **(U)**, miR-651 following initial analysis **(V)** and miR-891a following initial analysis **(W)**. MiRNAs with a *p* < 0.05 and an AUC > 0.8 were selected as successful distinguishing markers between tumor and normal tissues. Where miRLink; Initial Analysis, All Data; Meta-Analysis.

ROC analysis of all miRNAs in each tumor type, using the microarray expression data, evaluated the extent to which they could separate each tumor entity from the control group. The miRNAs with a *p* < 0.05 and an AUC > 0.8 were selected as successful distinguishing markers between each tumor type and the control group (Figure 5). More specifically, following the initial analysis the ROC curves yielded the following AUCs: a) For MBs and control group; miR-192 (AUC = 0.841, up-regulated) (Figure 5C), b) For AT/RTs and control group; miR-34a (AUC = 0.9, up-regulated) (Figure 5J). At the same time, the meta-analysis confirmed most of the identified miRNAs. In particular, miR-34a (AUC = 0.911) (Figure 5A) once again appeared to discriminate controls from embryonal tumors.

### Gene ontology and pathway analysis

**Figure 6:**
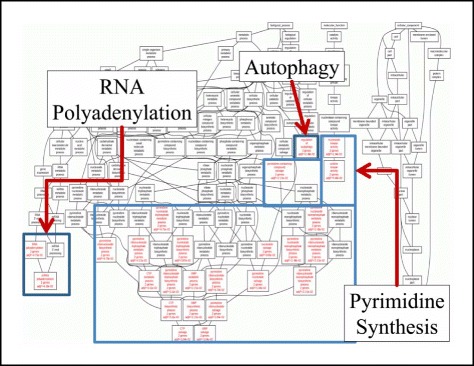
**GO annotation of the complete dataset.** Three main functions were revealed: RNA polyadenylation, Autophagy and Pyrimidine synthesis and processing.

Gene ontology analysis revealed that DE miRNAs obtained from meta-analysis participated in three main functions: RNA polyadenylation indicating a RNA processing role, Autophagy implying a metabolic and probably survival role and pyrimidine synthesis indicating proliferation (Figure 6). At the same time, pathway analysis did not show any significant pathway participation. Yet, disease association analysis revealed that these DE miRNAs participated in neoplasmatic diseases and neoplasm invasiveness (Additional file 4: Table S4).

## Discussion

Previous research in various cancer disease processes suggested that miRNAs play vital roles in disease pathogenesis and have potential as biomarkers and therapeutic agents. In particular, the use of miRNAs as embryonal tumor markers constitutes an exciting field in biomedical research, due to diverse aspects including ability to predict disease before the onset of clinical symptoms, rich information content, great discriminatory power, accessibility in different specimen types, possibility of being evaluated from different sources and their potential for accurate quantification following the application of high-throughput technologies [30]. Yet, principally due to the fact that the CNS is the least accessible of all tissues and the lack of clinically related animal models for miRNA research, no embryonal tumor miRNA biomarkers have been established in clinical practice [30,31].

In the current setting we performed miRNA microarrays in pediatric patients with embryonal CNS neoplasms to investigate whether the differential expression of miRNA genes is associated with factors that envisage the clinical course of the disease. Ultimately, we aimed to identify and provide additional data regarding potential miRNA expression signatures relative to prognosis of pediatric MBs and AT/RTs, specifically. At present, there have been comparatively limited reports focusing on identifying miRNA biomarkers related to MB and AT/RT diagnostics and therapeutics [23,26,28,32]. To our knowledge, this is the first attempt aiding specifically to evaluate an association between miRNA expression patterns and disease progression or patient outcome in pediatric MBs and AT/RTs and this is the first attempt that used meta-analysis to validate findings.

By performing miRNA microarrays, we identified overall 113 differentially expressed miRNAs between embryonal tumors and the control cohort. Among them, 107 were tissue-specific and 6 miRNAs were common between the two tumor types. More specifically, miR-34a, miR-548j, miR-3191 and miR-3912 were found up-regulated in both MBs and AT/RTs when compared to the control group. Based on previous reports, miR-34a is considered a putative tumor suppressor gene in various types of cancers [33-38] including brain tumors [39-42]. MiR-34a regulates the expression of SIRT1 (silent information regulator 1), which functions as an oncogene by inactivating key tumor suppressor proteins such as the transcription factor p53 [43-45]. Its targets include factors required for cell cycle progression, anti-apoptotic proteins and proteins involved in invasion [46].

In the current study, almost all analyses and associations confirmed the up-regulation of miR-34a in the embryonal tumors group and in the MB group alone. Overexpression of miR-34a has been previously observed in gastric cancer [47] as well as in pediatric brain tumors including medulloblastomas [23], ependymomas [48] pilocytic astrocytomas [28] and in low- and high-grade astrocytomas [49]. More specifically, our meta-analyses using the qRT-PCR raw data from Ferretti et al. [23], revealed that miR-34a was up-regulated in the 34 patients with MB that they screened. Additionally, according to Costa et al. miR-34a was found up-regulated in all 34 pediatric ependymomas that they tested with qRT-PCR against all normal samples. In Birks et al. [28] report, it is also obvious that miR-34a was overexpressed in all four pilocytic astrocytomas that they compared against a total of eight normal pediatric brain specimens, using miRNA microarrays. In addition, our meta-analysis using their raw data revealed that miR-34a was also overexpressed in all four MBs that they screened with miRNA microarrays. Findings from Birks et al. [28] are also in line with those reported by Ruiz Esparza-Garrido et al. [49] who later demonstrated that miR-34a was up-regulated in all twelve pediatric astrocytomas (3 pilocytic astrocytomas, 4 diffuse astrocytomas and 5 glioblastoma multiforme astrocytomas) they screened and compared to four control brain tissues. Bearing in mind our miR-34a findings, which a) have been validated with both additional experimentation and meta-analyses using more miRNA microarrays datasets and b) are in line with all aforementioned previous reports, it seems likely that miR-34a might not play global tumor suppressor roles. For reliable conclusions to be drawn this should be validated in even larger patient cohorts.

Using a strict filtering approach in the ROC tests, we determined a group of miRNAs that were also validated in an independent patient cohort. Therefore, we suggest that these miRNAs could be successfully used as distinguishing markers for each tumor type or between tumor types and normal tissues. It is noteworthy, that among them, once again, miR-34a was observed. In addition, we showed that miR-548j, and miR-3912, which have already manifested differential expression between embryonal tumors and the control cohort, have a high potential to distinguish AT/RTs alone from normal tissues, affording significant potential diagnostic value for AT/RTs. The role of miR-548j and miR-3912 is described for first time in embryonal tumors, as no previous related reports were found. Moreover, we observed that miR-192 and miR-3617 could accurately discriminate MBs over the normal tissues indicating their potential as putative biomarkers for MB diagnostics.

Overexpression of miR-192 contributes to tumor growth and progression in pancreatic ductal adenocarcinoma [50], and has been associated with early diagnosis of distant metastasis of gastric cancer [51], and with clinical relevance and prognostic significance in colon cancer [52]. Regarding brain tumors, our results are in line with Ruiz-Esparza-Garrido et al. [49], who manifested that miR-192 was up-regulated in 5 high-grade astrocytomas, when compared to 7 low-grade ones. On the contrary, though, Liu et al. [53] and Ferretti et al. [23] suggested that miR-192 was found significantly down-regulated in eight pediatric gliomas and in 34 pediatric MBs, respectively, whilst other reports in pediatric brain tumors have not identified this gene as differentially expressed [28,29,48,49,54]. Given these, the role of miR-192 in childhood CNS malignancies remains to be elucidated. No reports were found regarding the value of miR-3617 in similar study groups.

Hierarchical clustering analysis using the miRNA signatures revealed that AT/RTs formed distinct group versus the controls, therefore assisting in the diagnosis of this type of neoplasias. However, this was not demonstrated in MBs. We would agree with Sredni et al. [32] that miRNA expression profiling might not afford the finest tool to classify brain tumors for diagnostic purposes, especially in studies with small patient cohorts. Additional studies have also failed to cluster absolutely correct (100%) brain tumors based on miRNA expression patterns [24,25,28,55]. To validate this result, we have utilized an independent group of GE tumors. Germinomas are considered childhood tumors of embryonic origin, arising from primordial germ cells that have migrated aberrantly during embryonic development and subsequently undergone malignant transformation [56]. To investigate whether the inability of HCL was due to samples’ relativeness or other technical issues, we analyzed all data (MBs, AT/RTs, GEs) as a common cohort. Subsequently, HCL successfully discriminated between GEs and MBs or AT/RTs. This finding might indicate that MBs and AT/RTs are very close both in origin and in expression profiles. To our knowledge, miRNA expression has been found indisputably distinct between tumor types or tumors versus controls in one previous occasion [23].

The prognosis of early childhood medulloblastoma treated with surgery, radiotherapy and chemotherapy has been dismal (20%–45%) compared with older children, probably due to a diverse biology underlying medulloblastoma in younger children [57]. In addition, whether the molecular alterations described in embryonal tumors of older children are consistent in infants remain to be confirmed [7]. For this purpose we divided our patients to different age groups based on treatment intervention and we investigated potential distinct miRNA expression profiles. Overall, 11 miRNAs were found aberrantly expressed. Among them, the most imperative miRNAs with higher levels of expression obtained in youngest children (Group A) and lower levels of expression in older children (Group C) included miR-720 and miR-3681. It is noteworthy, that 62.5% (5/8) of children remain alive in Group A, although none of the children in this Group received radiotherapy. In Group C, 57.1% (4/7) of patients deceased from the embryonal tumor. In this Group, all children have received intense radiotherapy in combination with chemotherapy and surgery. It seems likely, that overexpression of miR-720 and miR-3681 confers an additional advantage to patients at higher-risk, whereas down-regulation of these miRNAs reflects dismal prognosis to lower-risk groups of patients. No reports were found about the value of miR-720 and miR-3681 in pediatric CNS malignancies.

In the current study we also endeavored to identify gender-specific miRNAs that could potentially contribute susceptibility to embryonal brain malignancies. According to Pinheiro et al. [58] notwithstanding the value of the X-linked miRNAs remains to be elucidated, several miRNAs participate in cancer onset and progression, while male preponderance has been observed in diverse types of cancer. Nevertheless, among the differentially expressed miRNAs identified in this setting, none of them was X located.

In an attempt to ascertain which miRNA signatures are implicated in the progression and outcome of the disease, we performed correlations between the following groups: control tissue cohort and patient cohort including relapsed patients and patients in complete remission as well as control tissue cohort and patient cohort including deceased patients and patients that remain alive, respectively. Based on our findings, following both initial and meta-analyses, we report the up-regulation of miR-3681, miR-34a, miR-136, miR-26b and miR-192 in the patient group, subsequently leading to the conclusion that they might possess oncogenic activities. The expression profiles initially obtained for miR-320e and miR-642a were not validated with meta-analyses. In addition, it is noticeable that the overexpression of miR-34a that we detected in previous correlations was once more confirmed. This is in line with Costa et al*.* [48] who also found miR-34a highly overexpressed in ependymomas, however, according to their results miR-34a portends a more favorable prognosis, instead. Yet again, miR-192 was found up-regulated in the patient cohort, underlying the potential of this gene, which according to our findings it emerges not only as a putative diagnostic biomarker, but also as predictor for the likelihood of an inferior clinical outcome.

Moreover, from current associations, although initially miR-601 emerged as a putative oncogene, neither meta-analysis nor qRT-PCR confirmed this finding. Therefore, it seems likely that this gene has potential tumor suppressor properties and our initial miRNA microarrays analysis was inadequate to reveal this. Previous reports were found describing both oncogenic and tumor suppressor roles for miR-601. For instance, elevated expression levels for miR-601 have been previously reported in pediatric ependymomas versus the control cohort and in pediatric glioblastomas when compared to anaplastic astrocytomas [25,48]. On the contrary, miR-601 might serve as a novel biomarker for the clinical diagnosis of colorectal cancer when down-regulated [55,59]. Based on our results, we propose that miR-601 plays putative tumor suppressor roles in MBs and its up-regulation is subsequently associated with favorable prognosis. Also, it is probable that it exerts tissue specific roles.

Remaining in the same context, both initial and meta-analyses showed that miR-720, miR-891a, miR-3665, miR-3620, miR-382, miR-452, and miR-122 were down-regulated in the patient group and therefore indicating that they might possess tumor-suppressive activities. Previous reports also proposed a possible link between these tumor-suppressor miRNA genes with diverse types of malignancies, on several occasions. For instance, reduced levels of miR-720 and miR-382 have been hitherto demonstrated in breast cancer [60,61] and MBs [23], respectively. Down-regulation of miR-452 has been associated with promoting stem-like traits and tumorigenesis of gliomas and it has been suggested that it may represent a novel prognostic biomarker and therapeutic target for the disease [62]. In addition, miR-122, has been well-documented to act as tumor-suppressor gene for hepatocellular carcinoma [63,64] and breast cancer [65]. Regarding brain tumors, under expression of miRNA-122 has been demonstrated in glioma specimens and glioma cell lines and has been inversely correlated with patients’ survival following surgery [66].

It is noteworthy, that the vast majority of the aforementioned miRNA oncogenes and tumor-suppressor genes were also observed when comparisons were made between miRNA expression patterns and the disease outcome, which verifies and validates their putative predictive role, sometimes favorable and sometimes poor, in embryonal CNS malignancies. These results were also confirmed by the regression analysis, which manifested both differential expressions in those miRNAs as well as linear behavior between tumor types.

Functional annotation of the miRNA cohort indicated that the DE miRNAs participate in RNA polyadenylation, which includes RNA processing, autophagy and pyrimidine synthesis. To the best of our knowledge, there are no reports available regarding such associations. It could be hypothesized that the identified miRNA cohort plays a role in tumor progression, since autophagy has been reported to be a survival mechanism in tumor cells [67]. At the same time, RNA processing and nucleotide synthesis are also signs of progressive disease, which probably could be the case for the present miRNA cohort. Interestingly, the identified miRNAs appeared to be of importance in tumors as signified by the *Disease Association* annotation analysis performed.

In summary, the current study was shown to provide significant insights into the growing role of several miRNA signatures in pediatric embryonal CNS neoplasms including MBs and AT/RTs. Following associations with certain patients’ clinical characteristics and cross-validation with meta-analyses, there was good evidence that miR-601 emerged as a putative tumor suppressor gene in MBs, whereas miR-34a manifested potential oncogenic roles. This is the first study to report the adverse properties of miR-34a as demonstrated in pediatric embryonal brain neoplasms, indicating tissue-specific roles rather than global tumor suppressor properties. Collectively, our findings modulated novel molecular biomarkers which might have a promising potential in pediatric embryonal CNS malignancies.

## Methods

### Patients and tumor samples

#### Initial analysis

Overall, 19 resected embryonal tumors were studied from children diagnosed with MBs (n = 15) or AT/RTs (n = 4) diagnosed according to the 2007 WHO criteria [68]. Specifically, the patient cohort included 8 males and 11 females, aged from 0.03 to 16.06 years (mean 4.56 ± 3.16 years). The median age of MB patients was 7.95 years with a male/female ratio of 1:1, the median age for the AT/RT patients was 1.1 years with a male/female ratio of 1:3, and the median age of the non-malignant cohort was 9 years with a male/female ratio of 1:0. Among the patients’ cohort, 9 patients (47.4%) remain in complete remission; 7 MB patients and 2 AT/RT patients, and 10 patients (52.6%) succumbed from the disease; 8 MB patients and 2 AT/RT patients. As controls, 13 samples were used; The First-Choice Human Brain Reference RNA was used (Ambion, Austin, TX, USA) and 12 samples were obtained from four deceased children who underwent autopsy and were not present with any brain distortion, including the following anatomic locations: cerebellum (n = 3), medulla oblongata (n = 3), parietal lobe (n = 3) and temporal lobe (n = 3).

**Figure 7:**
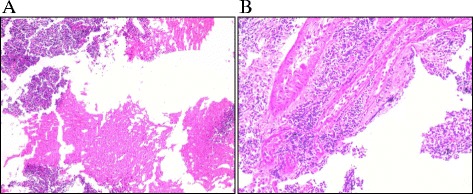
**Representative haematoxylin and eosin (H&E) staining from a non-malignant pediatric brain (cerebellum) (A) and an MB brain tumor (B).**

Haematoxylin and eosin (H&E) staining was performed in all embryonal brain tumor specimens (Figure 7). All samples were snap-frozen during resection and stored at −80°C until use. Clinicopathologic information such as age, tumor location, disease progression and survival for each specimen were collected by retrospective medical record review. The time of death was not available for the majority of patients and as a result, the estimation of overall survival was not feasible. All samples are summarized in Tables 2, 3 and presented thoroughly in Additional file 2: Table S1. The present study was conducted with the approval of “Aghia Sophia” Children’s Hospital Ethics Committee (Protocol No. 35/19. 16/09/13).

#### Meta-analyses

In the present study, for validation and further cross-analysis purposes, online available datasets; GSE19347^1^ [69] and GSE45126^2^ [70] and datasets from Birks *et al.* (2011) [28] were included. From the GSE19347 dataset, twelve germinoma (GE) samples were added as an independent validating dataset, whereas from the GSE45126 dataset, 100 control samples were included. Lastly, from Birks *et al.* (2011), four MBs, four ATRTs and eight control samples were added. Subsequently, in total, our meta-analysis included 39 CNS tumor samples; 19 MBs, 8 ATRTs, 12 GEs (not embryonal) and 121 control samples. All samples are summarized in Tables 2, 3 and presented thoroughly in Additional file 2: Table S1.

### MicroRNA profiling

In brief, total RNA and miRNAs were extracted using the Trizol standard protocol (Invitrogen, Carlsbad, CA) and the mirVANA miRNA isolation kit (Ambion, Austin, TX). The RNA quantity and quality were evaluated using a spectrophotometer (NanoDrop® ND-1000 UV–vis, Nanogen Inc.). Labelling and hybridization were performed using the LabelIT miRNA labelling kit (Mirus Bio LLC, USA) according to manufacturer’s instructions. Samples were hybridized to Applied MicroArrays (miRlink Bioarray 300054-3PK) platform. This array contained 1211 human miRNAs. Hybridization was performed at 37°C with rotation at 145 rpm for 16 h. Images were scanned using Agilent Microarray Scanner (G2565CA) controlled by Agilent Scan Control 7.0 software.

### Quantitative Real-Time Polymerase Chain Reaction (qRT-PCR) validation

Expression measurements of selected targets (mature miR-34a and miR-601) were studied in all tumor (*n* = 19) and control samples used in the initial miRNA microarray analysis and in an additional blinded independent set of 30 snap-frozen embryonal tumor samples (*n* = 21 MBs and *n* = 9 AT/RTs) using qRT-PCR. In brief, cDNA synthesis and subsequent RT-PCR were performed using a TaqMan MiRNA Reverse Transcription kit (Applied Biosystems, Inc., USA), and individual TaqMan MiRNA assays (Applied Biosystems, Inc., USA), according to the manufacturer’s recommendations. All samples were tested in triplicate in a LC480 LightCycler system (Roche GmbH, Switzerland). The RNU44 was used as endogenous control. Wells lacking template were used as negative controls. An additional online available miRNA microarray MB dataset from Feretti et al. was used, with GEO accession number GSE12303^3^ [23]. Expression changes were compared by relative quantification in the form of fold changes obtained with the ΔΔCt method.

### Statistical and data analyses

#### Microarray data extraction and pre-processing

The total gene signals were extracted using the Imagene 6.0 software (Biodiscovery Inc., USA) that contains summarized signal intensities for each miRNA by combining intensities of replicate probes and background subtraction. Raw data provided in the GEO database were used for meta-analysis. All data were extracted, pre-processed and sorted with Microsoft Excel®. Since each dataset had a cohort of miRNAs, we had to follow a common methodology for each dataset in order to obtain the maximum of common available miRNAs. This has been succeeded using the following method: Each dataset had a different number of replicates for each miRNA. Therefore we created a new matrix, which included all available samples (set as columns) and all available miRNAs (set as rows). This gave us a 19221 × 160 matrix, which included all miRNAs and all their respective replicates in all datasets. This matrix had numerous gaps, which were filled with the “NaN” value.

#### Microarray data analysis

The multiparameter analyses were performed with MATLAB® simulation environment (The Mathworks, Inc, Natick, MA). Since the final task was to calculate the mean of each miRNA replicate, the matrix was uploaded into the Matlab environment and “NaN” values were replaced as follows: for each miRNA the mean value was calculated in the respective dataset and NaNs were replaced by that value. This did not change the total distribution, while the mean of the miRNA remained the same. In that way, we have managed to avoid NaNs, which further on would interfere with our subsequent analysis. Next, probe replicates were combined and the mean was calculated, which was further used for analysis. Filtering was performed based on the signal intensity. Background correction was performed by subtracting the median local background from the signal intensity as previously reported [71]. Normalization was performed using the quantile normalization algorithm. The two tailed student t-test was used to test the mean differences between two groups. Continuous variables were expressed as median ± standard deviation unless differently indicated. MicroRNAs were considered to be significantly differentially expressed (DE) if they obtained a p-value < 0.05 and an FDR ≤ 0.05. Two-way average-linkage hierarchical clustering (HCL) with Euclidian distance was performed with MATLAB® software. The normalization and microarray statistical data analysis findings are presented in Additional file 9: Figure S1.

#### Group-wise comparison of miRNA expression and clinical data

MiRNA expression values were compared to clinical parameters including gender, diagnosis, brain location, tumor grade and developmental status (fetal, infancy, childhood, adolescence, survival). Differences were considered significant if they obtained a *p* < 0.05.

#### ROC analysis

Receiver Operating Characteristic (ROC) curves were established to evaluate the diagnostic value of differentially expressed miRNAs for differentiation between CNS tumors (MB, AT/RTs and GEs) and controls. ROC analysis was performed with the MATLAB® simulation environment (The Mathworks, Inc, Natick, MA). ROC analysis has been used in order to calculate Area Under the Curve (AUC), along with Standard Error (SE) and 95% Confidence Intervals (95% CI) [72]. ROC curves have been considered significant if they obtained an AUC value >0.8 and a *p*-value < 0.05.

#### MiRNA enrichment, gene ontology and pathway analysis

Differentially expressed miRNAs have been further enriched and analyzed for known functions and known pathway participation. This analysis was performed using the WebGestalt web-tool http://bioinfo.vanderbilt.edu/webgestalt/ [73,74].

## Endnotes

^1^http://www.ncbi.nlm.nih.gov/geo/query/acc.cgi?acc=GSE19347.

^2^http://www.ncbi.nlm.nih.gov/geo/query/acc.cgi?acc=GSE45126.

^3^http://www.ncbi.nlm.nih.gov/geo/query/acc.cgi?acc=GSE12303.

## Additional files

Additional file 1: Table S2. Common under- and up-regulated miRNAs between tumors following initial and meta-analyses as from *Venn* diagrams. Additional file 2: Table S1. Clinical Raw Data. Additional file 3: Figure S2. MicroRNA expression levels and patient age following initial analysis. Kruskal-Wallis analysis of miRNA expression profiles between patient age groups; Group A: < 3 years, Group B: 3–8 years and Group C: 9–18 years. Overall, 11 differentially expressed miRNAs were identified. Among them, 6 miRNAs were overexpressed in Group A as compared to the other groups: miR-1268 **(A)**, miR-3681 **(B)**, miR-3912 **(C)**, miR-601 **(D)**, miR-608 **(E)** and miR-720 **(F)**. Three miRNAs were up-regulated in Group B as compared to the other groups: miR-3665 **(G)**, miR-519c-3p **(H)** and miR-891a **(I)**. Finally, two miRNAs were up-regulated as compared to the other groups: miR-2052 **(J)** and miR-26b **(K)**. (*denotes a *p* < 0.05 significance and **denotes a *p* < 0.01 significance). Additional file 4: Table S4. Disease and Drug Association Annotations for DE miRNAs as predicted from enrichment analysis using WebGestalt web tool. Additional file 5: Figure S3. MicroRNA expression levels and patient gender following initial analysis. Kruskal-Wallis analysis of miRNA expression profiles between male and female patients within the miRLink dataset (in-house experiments). In total, 3 miRNAs were up-regulated in males miR-26b **(A)**, miR-3162 **(B)** and miR-1268 **(C)**, while 5 miRNAs were overexpressed in females; miR-720 (D), miR-186* (E), miR-3617 (F), miR-320c (G) and miR-3614-5p (H). (*denotes a p < 0.05 significance between pairs). Additional file 6: Table S3. DE miRNAs *log*_*2*_ transformed and natural mean expression values and statistical analysis between all clinical associations. Additional file 7: Figure S4. MicroRNA expression levels and disease progression following initial analysis. Kruskal-Wallis analysis between DE miRNAs and disease progression, following initial analysis; Relapse (n = 9) or Complete Remission (n = 10) (CR). Overall, 18 miRNAs were differentially expressed; 5 miRNAs were found up-regulated in the group of patients that are in complete remission when compared to the relapsed or the control groups; miR-3681 **(A)**, miR-601 **(B)**, miR-642a **(C)**, miR-136 **(D)** and miR-26b **(E)**. Additionally, three miRNAs were found up-regulated in relapsed patients when compared to the group of patients that are in Complete Remission (CR) or the control group; mIR-192 **(F)**, miR-320e **(G)** and miR-34a **(H)**. Finally, ten miRNAs were found overexpressed in the control group when compared to the patients group (relapsed or in Complete Remission (CR)); miR-720 **(I)**, miR-891a **(J)**, miR-522 **(K)**, miR-518c **(L)**, miR-3665 **(M)**, miR-891a **(N)**, miR-382 **(O)**, miR-452 **(P)**, miR-122 **(Q)**, miR-147 **(R)**. Additional file 8: Figure S5. MicroRNA expression levels and survival following initial analysis. Kruskal-Wallis analysis between DE miRNAs and patient outcome; alive (n = 9) or deceased (n = 10). In total, 8 miRNAs were significantly different; five miRNAs weres found up-regulated in alive patients when compared to the group of deceased and control samples. In particular, miR-3681 **(A)**, miR-642a **(B)**, miR-26b **(C)**, miR-136 **(D)** and miR-320e **(E)** were increased in alive samples as well as manifested linear regression with respect to expression moving from alive samples to controls. Two miRNAs were found down-regulated in the group of patients that remain alive when compared to the diseased or the control groups. In particular, miR-720 **(F)** and miR-891a **(G)** manifested similar linear regression increasing from alive samples to controls. Finally, one miRNA, miR-320c (H), manifested higher expression levels in deceased samples as compared to alive and control samples (* denotes a p < 0.05 significance). Additional file 9: Figure S1. Analysis results of microarray data. Quantile normalization **(A)**, T-test histograms **(B)**, Volcano plot of T-test results **(C)**, False Discovery Rate (FDR) of T-test results **(D)**.

## Abbreviations

CNS: Central nervous system
AT/RT: Atypical terathoid/rhabdoid tumor
MB: Medulloblastoma
FDR: False discovery rate
GE: Germinoma
DE: Differentially expressed
AUC: Area under the curve
ROC: Receiver operating characteristic
qRT-PCR: Quantitative real time reverse transcription polymerase chain reaction
HCL: Hierarchical clustering
miRNA: micro RNA
WHO: World Health Organization
H&E: Hematoxylin eosin
IHC: Immunohistochemistry
CR: Complete remission
RE: Relapse
SE: Standard error
CI: Confidence intervals
GO: Gene ontology
