# Supplementary material for: Microrna expression signatures predict patient progression and disease outcome in pediatric embryonal central nervous system neoplasms
Source: J Hematol Oncol. 2014 Dec 31;7:96. doi: 10.1186/s13045-014-0096-y (PMC4342799; doi:10.1186/s13045-014-0096-y)

**Supplementary Figure 2.** **MicroRNA expression levels and patient age following initial analysis.** Kruskal-Wallis analysis of miRNA expression profiles between patient age groups; Group A: ˂ 3 years, Group B: 3-8 years and Group C: 9-18 years. Overall, 11 differentially expressed miRNAs were identified. Among them, 6 miRNAs were overexpressed in Group A as compared to the other groups: miR-1268 (**A**), miR-3681 (**B**), miR-3912 (**C**), miR-601 (**D**), miR-608 (**E**) and miR-720 (**F**). Three miRNAs were up-regulated in Group B as compared to the other groups: miR-3665 (**G**), miR-519c-3p (**H**) and miR-891a (**I**). Finally, two miRNAs were up-regulated as compared to the other groups: miR-2052 (**J**) and miR-26b (**K**). (* denotes a *p*<0.05 significance and ** denotes a *p*<0.01 significance).


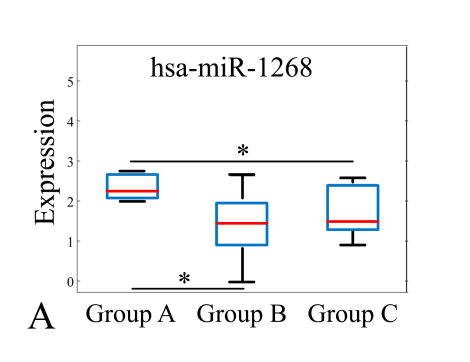

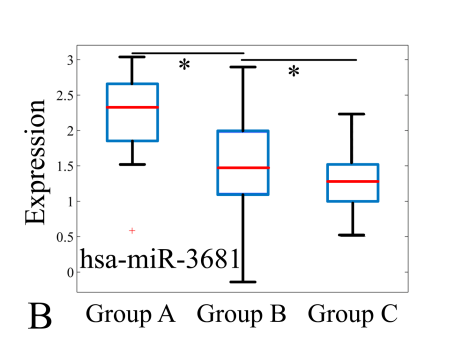

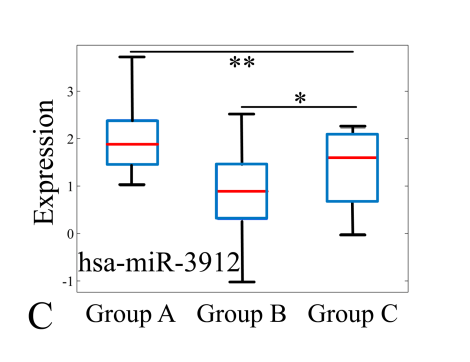

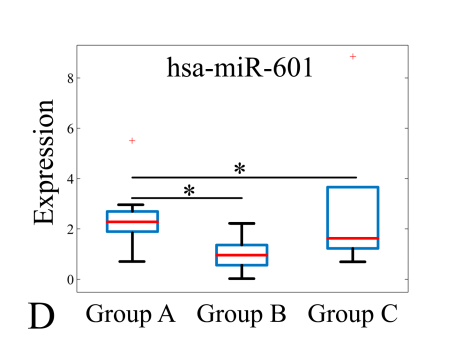

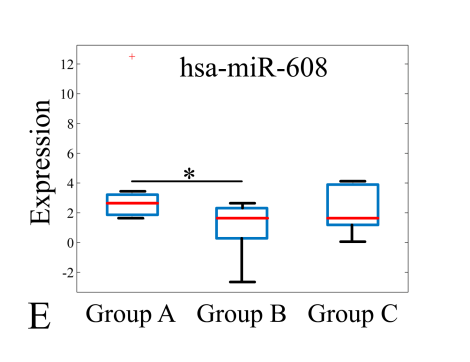

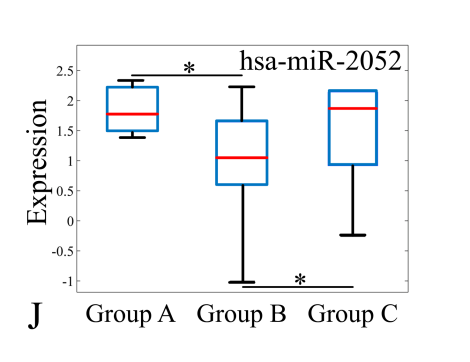

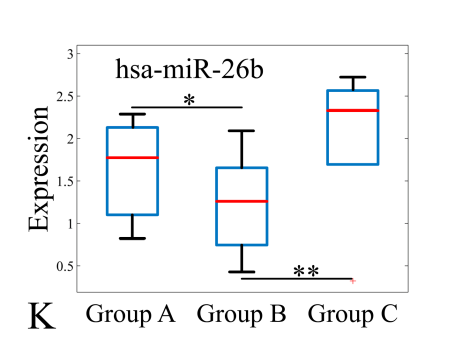

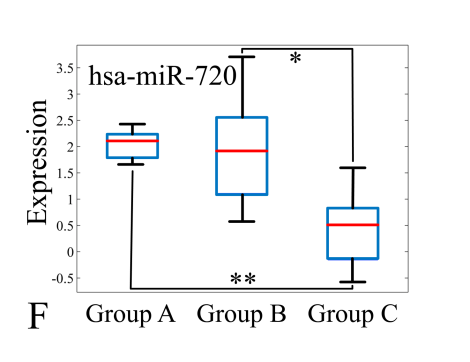

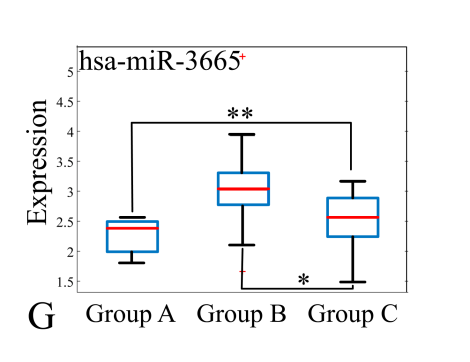

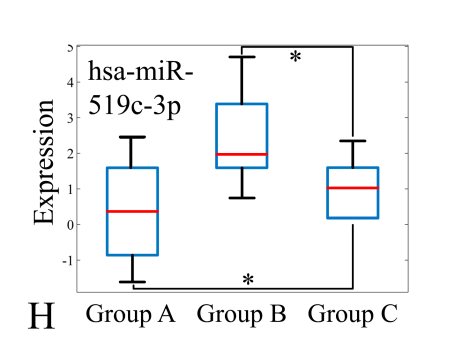

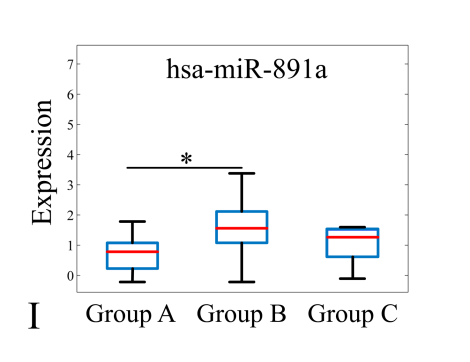

Supplement: Additional file 3: Figure S2. — MicroRNA expression levels and patient age following initial analysis. Kruskal-Wallis analysis of miRNA expression profiles between patient age groups; Group A: < 3 years, Group B: 3–8 years and Group C: 9–18 years. Overall, 11 differentially expressed miRNAs were identified. Among them, 6 miRNAs were overexpressed in Group A as compared to the other groups: miR-1268 (A), miR-3681 (B), miR-3912 (C), miR-601 (D), miR-608 (E) and miR-720 (F). Three miRNAs were up-regulated in Group B as compared to the other groups: miR-3665 (G), miR-519c-3p (H) and miR-891a (I). Finally, two miRNAs were up-regulated as compared to the other groups: miR-2052 (J) and miR-26b (K). (*denotes a p < 0.05 significance and **denotes a p < 0.01 significance). [file 13045_2014_96_MOESM3_ESM.docx]
