# Supplementary material for: Microrna expression signatures predict patient progression and disease outcome in pediatric embryonal central nervous system neoplasms
Source: J Hematol Oncol. 2014 Dec 31;7:96. doi: 10.1186/s13045-014-0096-y (PMC4342799; doi:10.1186/s13045-014-0096-y)

**Supplementary Figure 3. MicroRNA expression levels and patient gender following initial analysis.** Kruskal-Wallis analysis of miRNA expression profiles between male and female patients within the miRLink dataset (in-house experiments). In total, 3 miRNAs were up-regulated in males miR-26b (**A**), miR-3162 (**B**) and miR-1268 (**C**), while 5 miRNAs were overexpressed in females; miR-720 (**D**), miR-186* (**E**), miR-3617 (**F**), miR-320c (**G**) and miR-3614-5p (**H**). (* denotes a p<0.05 significance between pairs).


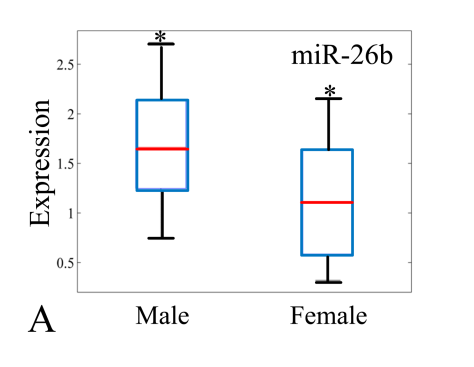

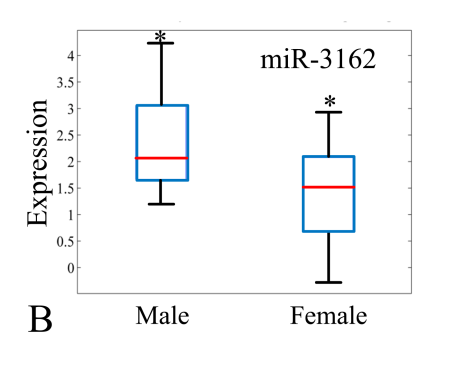

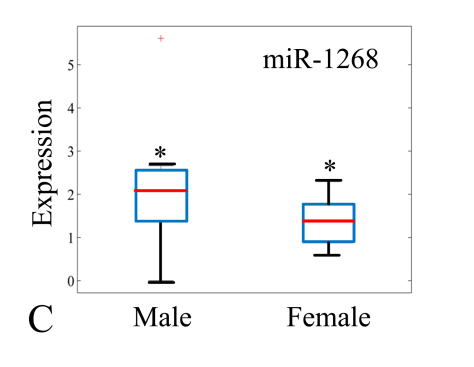

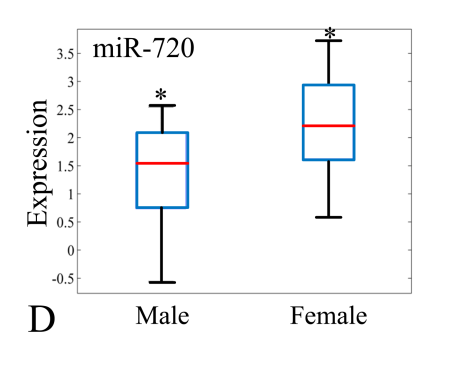

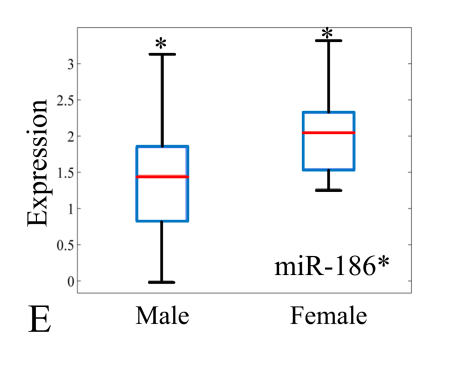

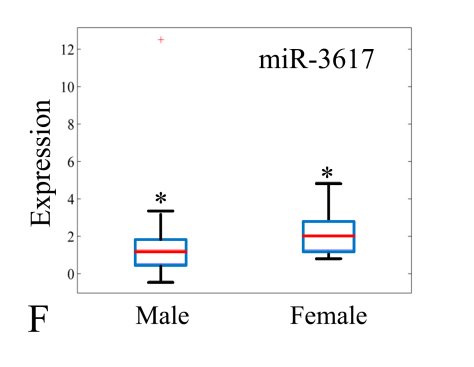

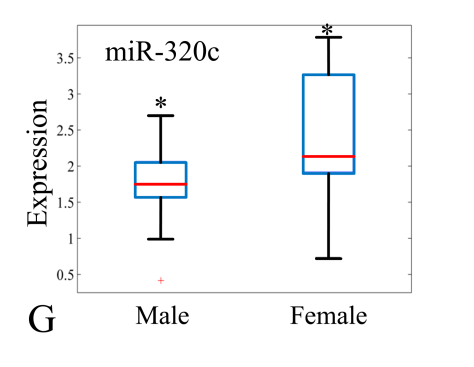

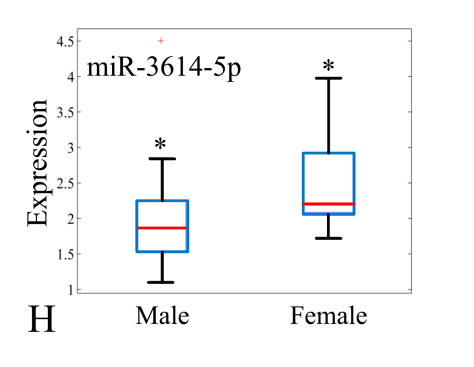

Supplement: Additional file 5: Figure S3. — MicroRNA expression levels and patient gender following initial analysis. Kruskal-Wallis analysis of miRNA expression profiles between male and female patients within the miRLink dataset (in-house experiments). In total, 3 miRNAs were up-regulated in males miR-26b (A), miR-3162 (B) and miR-1268 (C), while 5 miRNAs were overexpressed in females; miR-720 (D), miR-186* (E), miR-3617 (F), miR-320c (G) and miR-3614-5p (H). (*denotes a p < 0.05 significance between pairs). [file 13045_2014_96_MOESM5_ESM.docx]
