# Supplementary material for: Microrna expression signatures predict patient progression and disease outcome in pediatric embryonal central nervous system neoplasms
Source: J Hematol Oncol. 2014 Dec 31;7:96. doi: 10.1186/s13045-014-0096-y (PMC4342799; doi:10.1186/s13045-014-0096-y)

**Supplementary Figure 4. MicroRNA expression levels and disease progression following initial analysis.** Kruskal-Wallis analysis between DE miRNAs and disease progression, following initial analysis; Relapse (n=9) or Complete Remission (n=10) (CR). Overall, 18 miRNAs were differentially expressed; 5 miRNAs were found up-regulated in the group of patients that are in complete remission when compared to the relapsed or the control groups; miR-3681 (**A**), miR-601 (**B**), miR-642a (**C**), miR-136 (**D**) and miR-26b (**E**). Additionally, three miRNAs were found up-regulated in relapsed patients when compared to the group of patients that are in Complete Remission (CR) or the control group; mIR-192 (**F**), miR-320e (**G**) and miR-34a (**H**). Finally, ten miRNAs were found overexpressed in the control group when compared to the patients group (relapsed or in Complete Remission (CR)); miR-720 (**I**), miR-891a (**J**), miR-522 (**K**), miR-518c (**L**), miR-3665 (**M**), miR-891a (**N**), miR-382 (**O**), miR-452 (**P**), miR-122 (**Q**), miR-147 (**R**).


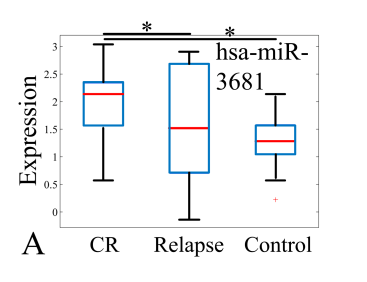

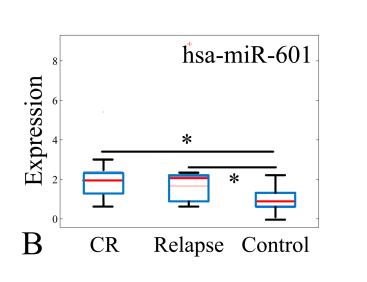

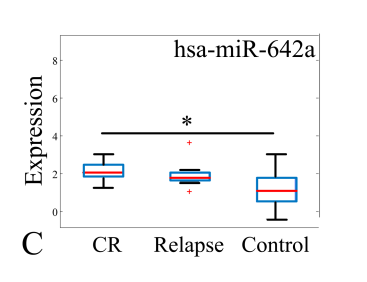

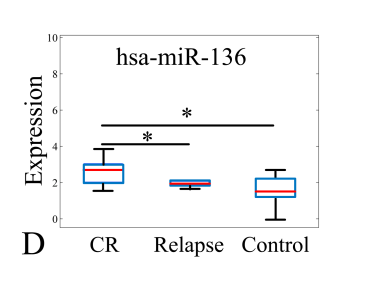

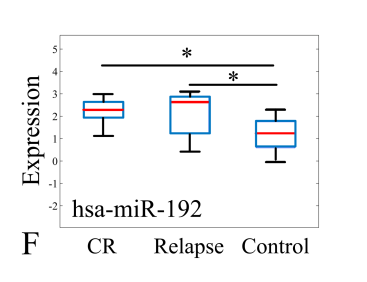

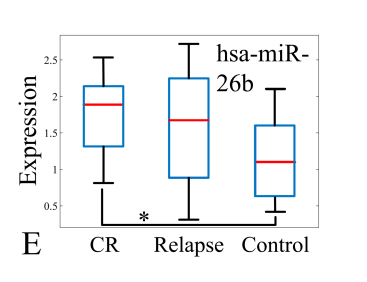

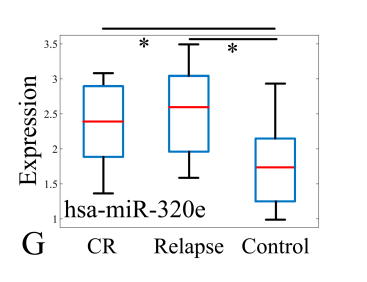

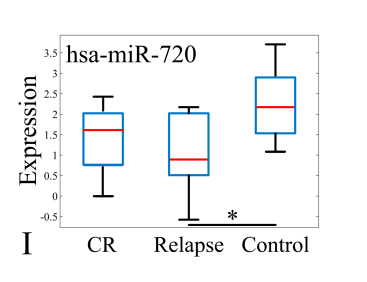

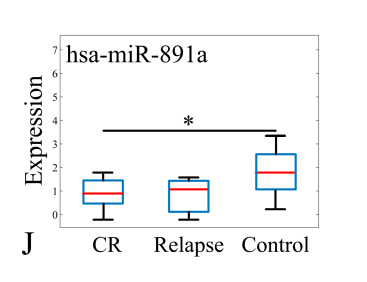

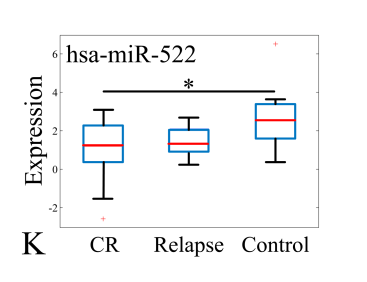

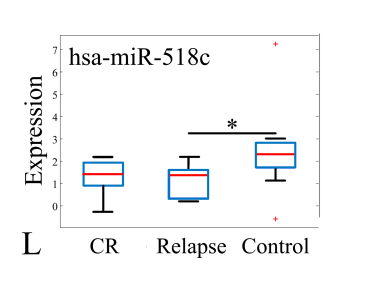

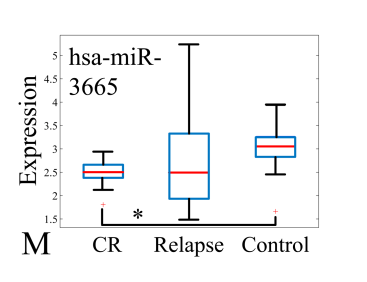

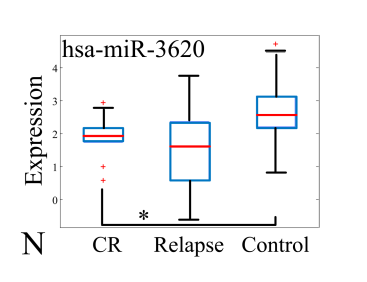

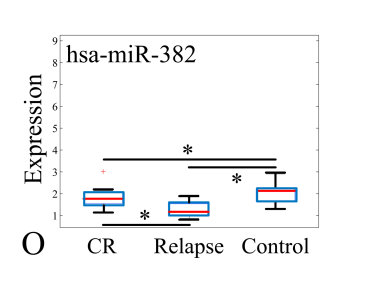

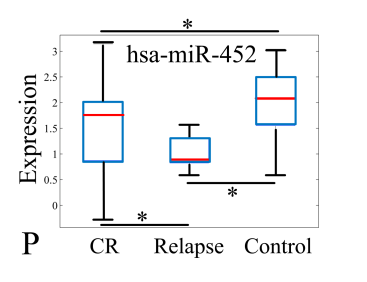

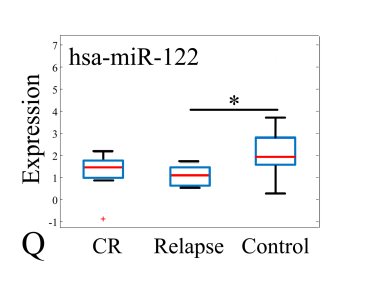

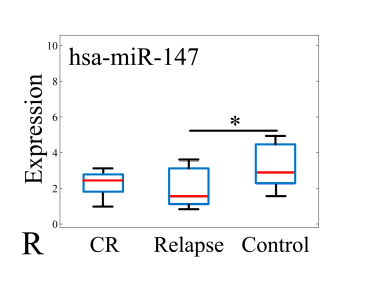

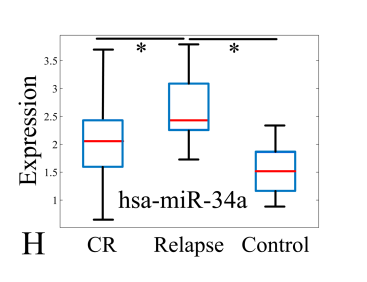

Supplement: Additional file 7: Figure S4. — MicroRNA expression levels and disease progression following initial analysis. Kruskal-Wallis analysis between DE miRNAs and disease progression, following initial analysis; Relapse (n = 9) or Complete Remission (n = 10) (CR). Overall, 18 miRNAs were differentially expressed; 5 miRNAs were found up-regulated in the group of patients that are in complete remission when compared to the relapsed or the control groups; miR-3681 (A), miR-601 (B), miR-642a (C), miR-136 (D) and miR-26b (E). Additionally, three miRNAs were found up-regulated in relapsed patients when compared to the group of patients that are in Complete Remission (CR) or the control group; mIR-192 (F), miR-320e (G) and miR-34a (H). Finally, ten miRNAs were found overexpressed in the control group when compared to the patients group (relapsed or in Complete Remission (CR)); miR-720 (I), miR-891a (J), miR-522 (K), miR-518c (L), miR-3665 (M), miR-891a (N), miR-382 (O), miR-452 (P), miR-122 (Q), miR-147 (R). [file 13045_2014_96_MOESM7_ESM.docx]
