# Supplementary material for: Microrna expression signatures predict patient progression and disease outcome in pediatric embryonal central nervous system neoplasms
Source: J Hematol Oncol. 2014 Dec 31;7:96. doi: 10.1186/s13045-014-0096-y (PMC4342799; doi:10.1186/s13045-014-0096-y)

**Supplementary Figure 5. MicroRNA expression levels and survival following initial analysis.** Kruskal-Wallis analysis between DE miRNAs and patient outcome; alive (n=9) or deceased (n=10). In total, 8 miRNAs were significantly different; five miRNAs were found up-regulated in alive patients when compared to the group of deceased and control samples. In particular, miR-3681 (**A**), miR-642a (**B**), miR-26b (**C**), miR-136 (**D**) and miR-601 (**E**) were increased in alive samples as well as manifested linear regression with respect to expression moving from alive samples to controls. Two miRNAs were found down-regulated in the group of patients that remain alive when compared to the diseased or the control groups. In particular, miR-720 (**F**) and miR-891a (**G**) manifested similar linear regression increasing from alive samples to controls. Finally, one miRNA, miR-320c (**H**), manifested higher expression levels in deceased samples as compared to alive and control samples (* denotes a p<0.05 significance).


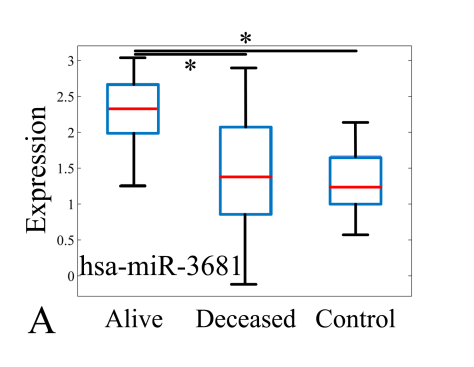

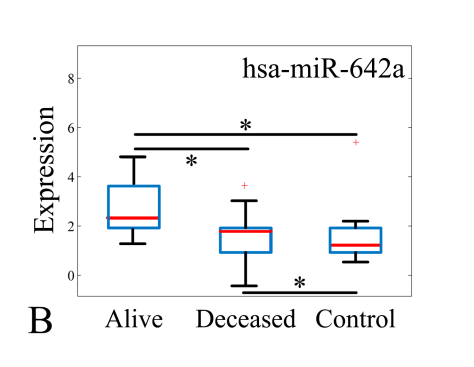

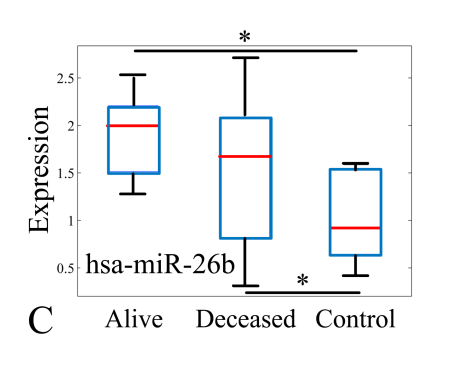

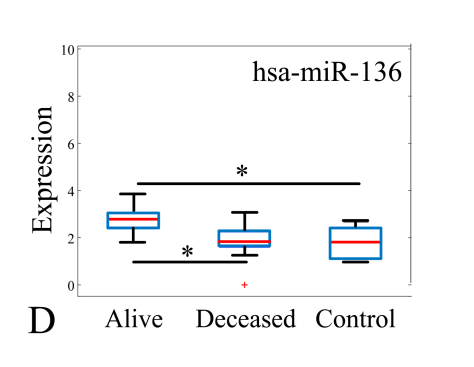

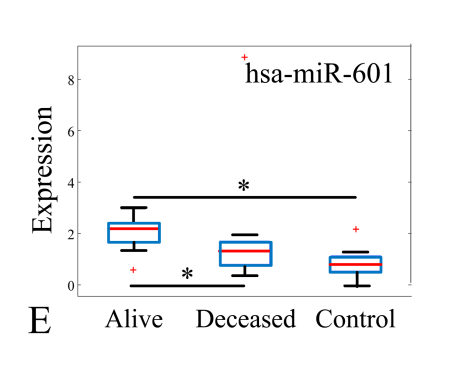

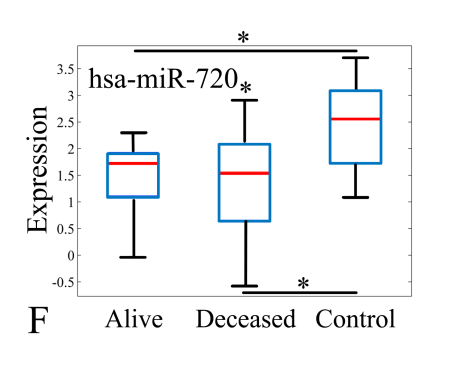

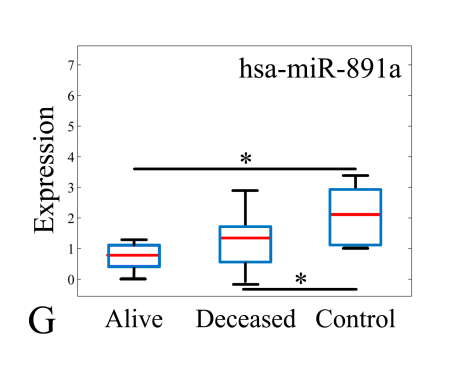

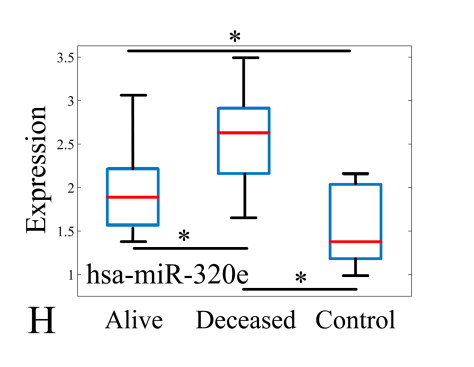

Supplement: Additional file 8: Figure S5. — MicroRNA expression levels and survival following initial analysis. Kruskal-Wallis analysis between DE miRNAs and patient outcome; alive (n = 9) or deceased (n = 10). In total, 8 miRNAs were significantly different; five miRNAs weres found up-regulated in alive patients when compared to the group of deceased and control samples. In particular, miR-3681 (A), miR-642a (B), miR-26b (C), miR-136 (D) and miR-320e (E) were increased in alive samples as well as manifested linear regression with respect to expression moving from alive samples to controls. Two miRNAs were found down-regulated in the group of patients that remain alive when compared to the diseased or the control groups. In particular, miR-720 (F) and miR-891a (G) manifested similar linear regression increasing from alive samples to controls. Finally, one miRNA, miR-320c (H), manifested higher expression levels in deceased samples as compared to alive and control samples (* denotes a p < 0.05 significance). [file 13045_2014_96_MOESM8_ESM.docx]
