# Supplementary material for: Microrna expression signatures predict patient progression and disease outcome in pediatric embryonal central nervous system neoplasms
Source: J Hematol Oncol. 2014 Dec 31;7:96. doi: 10.1186/s13045-014-0096-y (PMC4342799; doi:10.1186/s13045-014-0096-y)
Supplement: Additional file 9: Figure S1. — Analysis results of microarray data. Quantile normalization (A), T-test histograms (B), Volcano plot of T-test results (C), False Discovery Rate (FDR) of T-test results (D). [file 13045_2014_96_MOESM9_ESM.docx]

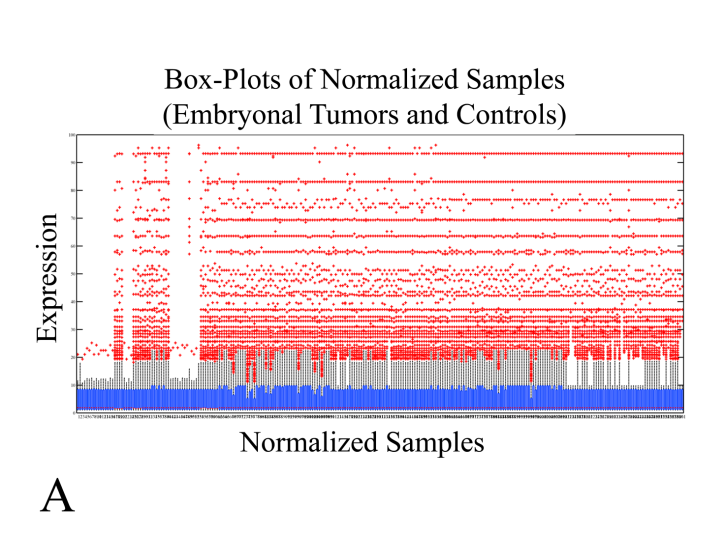

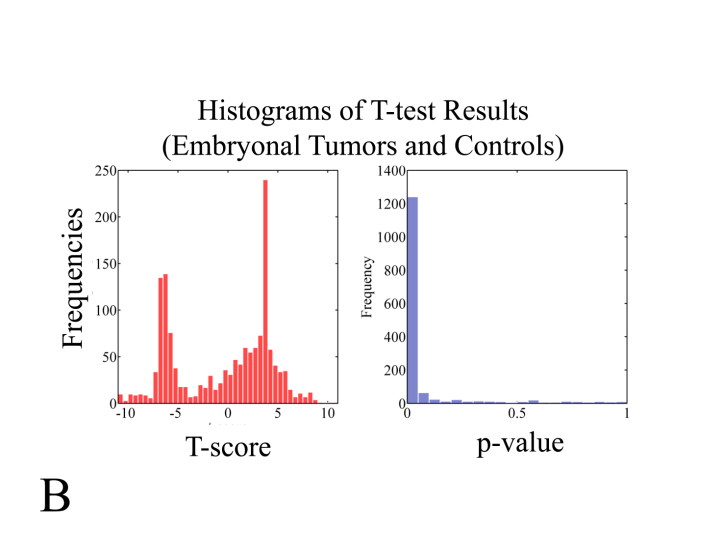

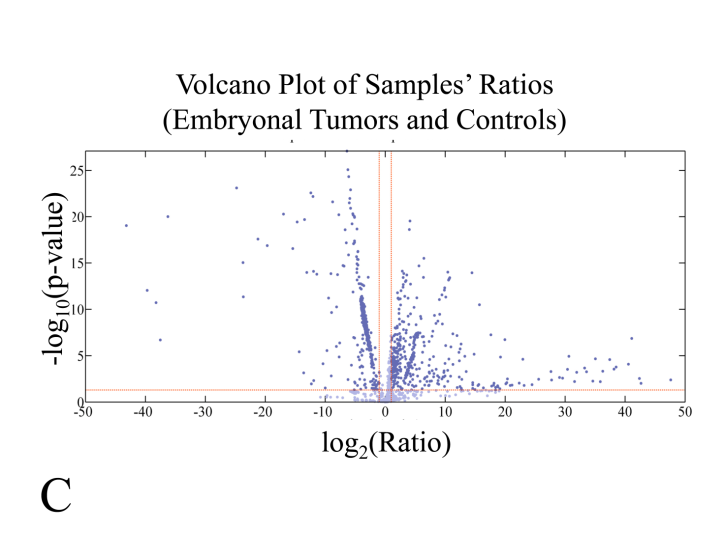

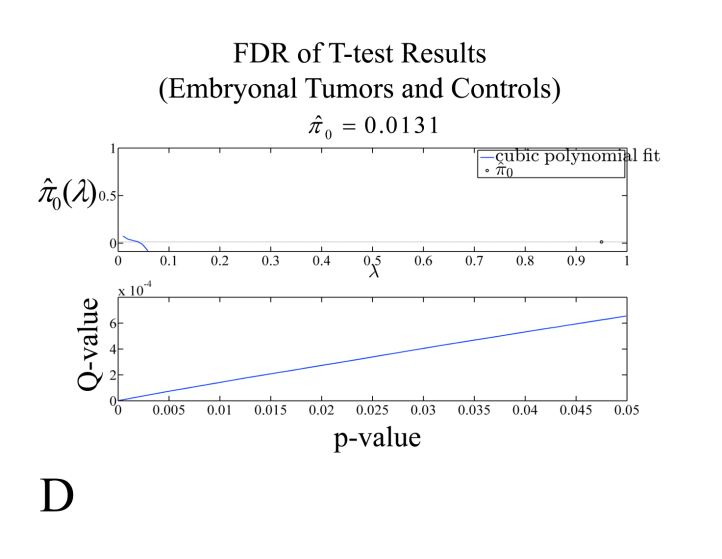


**Supplementary Figure 1**. Analysis results of microarray data. Quantile normalization (**A**), T-test histograms (**B**), Volcano plot of T-test results (**C**), False Discovery Rate (FDR) of T-test results (**D**).
